# Effectiveness of PCR primers for the detection of occult hepatitis B virus infection in Mexican patients

**DOI:** 10.1371/journal.pone.0205356

**Published:** 2018-10-10

**Authors:** Francisca Sosa-Jurado, Daniel Meléndez-Mena, Nora H. Rosas-Murrieta, Belinda Guzmán-Flores, Miguel A. Mendoza-Torres, Roberto Barcenas-Villalobos, Luis Márquez-Domínguez, Paulina Cortés-Hernández, Julio Reyes-Leyva, Verónica Vallejo-Ruiz, Gerardo Santos-López

**Affiliations:** 1 Laboratorio de Biología Molecular y Virología, Centro de Investigación Biomédica de Oriente, Instituto Mexicano del Seguro Social, Metepec, Atlixco, Puebla, México; 2 Servicio de Gastroenterología, Unidad Médica de Alta Especialidad, Centro Médico Nacional "General de División Manuel Ávila Camacho", Instituto Mexicano del Seguro Social, Puebla, Puebla, México; 3 Centro de Química, Instituto de Ciencias, Benemérita Universidad Autónoma de Puebla, Puebla, Puebla, México; 4 Banco de Sangre, Unidad Médica de Alta Especialidad, Centro Médico Nacional "General de División Manuel Ávila Camacho", Instituto Mexicano del Seguro Social, Puebla, Puebla, México; 5 Laboratorio de Biología Celular, Centro de Investigación Biomédica de Oriente, Instituto Mexicano del Seguro Social, Metepec, Atlixco, Puebla, México; Centre de Recherche en Cancerologie de Lyon, FRANCE

## Abstract

**Background:**

Occult hepatitis B infection (OBI) is defined as the presence of hepatitis B virus (HVB) DNA in the liver of HBsAg negative individuals with or without detectable viral DNA in serum. OBI is a diagnostic challenge as it is characterized by a very low viral load, intermittently detectable through time. Individuals with OBI can develop chronic hepatic disease, including liver cirrhosis and hepatocellular carcinoma. The aim of this work was to produce tools to improve OBI detection of the HVB genotypes prevalent in Mexico.

**Methods:**

We designed and tested primers to detect OBI in serum samples by nested and real-time PCR. Conserved sites in the viral genome were determined by alignment of the most frequent HBV genotypes in Mexico (H, G/H, F and D) and primers spanning the entire viral genome were designed for first round and nested PCR. Primers were tested in serum samples of 45 patients not co-infected with hepatitis C virus or with HIV, out of a group of 116 HBsAg (-)/anti-HBc (+) individuals. Primers were also tested in a control group with chronic HBV. Nested PCR products obtained from HBsAg (-)/anti-HBc (+) were sequenced and used to design primers for real-time PCR (SYBR Green).

**Results:**

The most effective primer pairs to detect HBV products by nested PCR targeted ORF regions: PreS_2_/P, S/P, X/PreC, and C; while by real-time PCR they targeted ORF regions PreS_2_/P, S/P, X, and C. Out of the 45 HBsAg (-)/anti-HBc (+) patients tested, the viral genome was detected in 28 (62.2%) and 34 (75.5%), with nPCR and real-time PCR respectively.

**Conclusion:**

Primers designed for real-time PCR detected up to 75.5% of suspected OBI Mexican patients, with or without liver disease, which represents an improvement from previous PCR strategies.

## Introduction

Hepatitis B virus (HBV) is the most prevalent and dangerous infectious agent among those that produce hepatic disease in humans [1]. The main serologic markers used for screening in transfusion medicine are the surface antigen (HBsAg) and specific antibodies against the core protein (anti-HBc); however, for a more effective diagnosis the e-antigen (HBeAg), anti-HBsAg (anti-HBs) and viral DNA detection may be included [2]. Occult hepatitis B virus infection (OBI) is defined as the presence of HBV DNA in the liver (with or without detectable HBV DNA in serum) in HBsAg negative individuals [3] and it is the most complex to diagnose HBV infection [4].

Even though manufacturers have improved commercial HBsAg detection assays to include the most prevalent HBsAg mutants and to increase sensitivity [5–7], failures in diagnosis due to amino acid changes in HBsAg still occur. Mutations in the so-called "a" determinant at the center of the S ORF or in adjacent regions have been related to the escape from antibodies generated by natural infection or by vaccination and are involved in HBV diagnostic failures [8–10]. The viral DNA in occult hepatitis B virus infection is able to replicate, transcribe and synthesize proteins at very low levels and this infection may lead to the development of severe forms of liver disease [11].

Currently there is no standard assay for OBI diagnosis in liver tissue or serum and the only consistent method is the detection of viral DNA by real-time PCR or nested PCR (nPCR). Real-time PCR gives better results than nested PCR, but both techniques depend on the annealing capacity of specific primers on the circulating HBV genotypes [3]. The detection of both HBsAg and viral DNA is a diagnostic challenge of particular relevance for OBI diagnosis. In the present study, we determined the efficacy of primer sets designed to anneal in different sites of the viral genome to amplify diverse segments along the HBV genome. These primers were used to detect viral DNA in HBsAg (-)/anti-HBc (+) patients.

## Material and methods

### Patients

The study was carried out in accordance with the Declaration of Helsinki and in accordance with ethical regulations approved by the institutional ethics committee (Comité Local de Investigación en salud 2101 IMSS; study number R-2010-2101-31). Patients were invited to participate at the Gastroenterology Service of the National Health Centre, Manuel Avila Camacho, Instituto Mexicano del Seguro Social (IMSS) in Puebla City, Mexico. A total of 116 HBsAg (-)/anti-HBc (+) patients were identified in the Gastroenterology Service between June 2013 and December 2015. Among these, patients with the following criteria were included in the study: female or male, >18 years old, with at least three similar previous HBsAg (-)/anti-HBc (+) results in the last 5 years, not co-infected with HCV or with HIV, no history of treatment for HBV, HCV or HIV, not transplanted or transfused in the last 10 years, willing to participate and to sign a written informed consent letter.

For the analysis, we considered as occult hepatitis B patients the HBsAg (-) subjects with HBV DNA detected in at least three different viral genomic regions, using nested PCR or real-time PCR and with at least one amplicon confirmed by Sanger sequencing.

### Control group

Serum samples of 8 blood donors (BD) HBsAg (+)/anti-HBc (+)/HBV DNA (+), not coinfected with HCV and/or HIV were included as positive HBV controls. Additionally, two blood donors that were HBsAg (-)/anti-HBc (+)/HBV DNA (+) and HBsAg (-)/anti-HBc (-)/HBV DNA (+), were included and analyzed along the controls.

### Detection of serological markers

All serum samples of patients and controls were analyzed for HBsAg, anti-HBc, anti-HCV and anti-HIV/Agp24 serological markers, using a chemiluminescent microparticle immunoassay (CMIA, Abbot Laboratories Diagnostics Division, Abbott Park, IL, USA). All HBsAg (-)/anti-HBc (+) sera were further explored for detection of HBeAg (EIA 3890) and Anti-HBeAg (EIA 3891) by Enzyme Immunoassay (EIA; DRG International Inc., Springfield, NJ, USA).

### Automated detection of viral nucleic acids

Samples of blood donors were analyzed for detection of HIV-1 RNA, HCV RNA, and HBV DNA by the Procleix Ultrio Plus System (Gen-Probe Incorporated, San Diego, CA, USA), that screens for the genetic material of all three viruses simultaneously. The particular viruses present in screened samples (HIV-1, HCV, and/or HBV) were further detected by the Procleix Ultrio Plus Discriminatory Assays (Gen-Probe Incorporated, San Diego, CA, USA). All control individuals included in this study had HBV DNA detectable by the Procleix Ultrio Plus System, except the blood donor that was HBsAg (-)/anti-HBc (+), who was negative in Procleix assays and was identified as HBV DNA(+) by PCR (as described below).

### Primer design for PCR assays

Primer pairs to amplify fragments of the hepatitis B genome covering the PreS_1_, PreS_2_, S, P, X, PreC and Core regions, were designed with Primer 3 v.0.4.0 software (http://bioinfo.ut.ee/primer3-0.4.0/) [12]. The conserved sites in these regions were determined by alignment of complete genome sequences of the most frequent genotypes in Mexico, (ClustalW2, https://www.ebi.ac.uk/Tools/msa/clustalw2/help/faq.html) which are mainly H and G/H; but some D and F have also been reported in relation to occult hepatitis B virus cases.

We predicted amplicons ranging between 454 and 1158 bp in first round (1R) PCRs, while one or more amplicons ranging between 236–644 bp were predicted for second round PCR (nested PCR). Primers for real-time PCR (SYBR Green) were designed considering the HBV sequences obtained from nested PCR in this work, using Primer Quest Tool software (Integrated DNA Technologies Inc., Commercial Park Coralville, IA 52241, USA) [13]. All primers were synthesized by Integrated DNA Technologies Inc. Primer sequences are described in S1 Table.

### DNA extraction

Total DNA in serum samples (1 ml) was obtained using the QIAamp UltraSens Virus Kit (Qiagen, Hilden, Germany) according to manufacturer's instructions. The sample from the blood donor in the control group (HBsAg (-)/anti-HBc (+)) that by the Procleix system had undetectable HBV DNA (BDOBI^1^), was first concentrated by polyethylene glycol precipitation [14] and the pellet was resuspended in HEPES-Tris / imidazole pH7.4 buffer, before DNA extraction with the kit.

### PCR standardization

A gradient thermocycler (PTC-200, MJ Research, Waltham, MA, USA) was used to obtain the optimal amplification conditions for each primer pair. Seven products of first round (1R) PCR (S1 Table) were used for the subsequent nested PCR reactions. The annealing temperature ranged from 45°C to 68°C, denaturation was at 95°C and extension at 72°C. PCR Master Mix (Promega, Madison, WI, USA) was used to perform both the first and nested PCRs using primers at 200 nM. Eight options to perform the nested PCR were designed and are shown in S1 Table. PCR products were visualized by 1% agarose gel electrophoresis with ethidium bromide. The Procleix Ultrio Tigris System and Cobas Amplicor DNA HBV (Roche Diagnostic, IN, USA) negative controls, as well as distilled water were included as quality controls in PCR.

### Cloning and sequencing of PCR products

The PCR products were cloned into the pJET1.2/blunt Cloning Vector (CloneJET PCR Cloning Kit, Thermo Scientific Inc) according to the manufacturer's instructions. Cloned products were sequenced with the GenomeLab Dye Terminator Cycle Sequencing Kit and the automatic sequencer GenomeLab GeXP Genetic Analysis System (Beckman-Coulter, Pasadena, CA, USA). Plasmids were used in subsequent assays as positive controls. For the rest of subjects, the uncloned fragments were sequenced with their respective primers.

The HBV genome sequences obtained from HBsAg (-)/anti-HBc (+) patients were submitted to GenBank (www.ncbi.nlm.nih.gov/genebank) and the following accession numbers were assigned: KY564282-KY564304, KY564305-KY564310, KY762267-KY762285, KY807557-KY807561, KY595528-KY595536 and KY595538-KY595540.

### Real-time PCR

Power SYBR Green PCR Master Mix (2X) (Applied Biosystems, Foster City CA, USA) or Maxima SYBR Green/ROX Master Mix (2X) (Thermo Scientific, MA, USA) were used to detect the HBV sequences by qualitative real-time PCR. Both Master Mix reagent kits were used in 10 μl reactions containing 150 to 200 nM of each primer (S1 Table) and 4 μl DNA. The amplification reaction was performed using the StepOne Real-Time PCR System (Applied Biosystems, Foster City, CA, USA). Cycling conditions were as follows: 10 min at 95°C, and 40 cycles of 15s at 95°C followed by 1 min at 54–60°C. A melting curve was performed in each assay. The real time PCR reactions were made by triplicate or quadruplicate.

## Results

### Effectiveness of HBV genome detection by 1R-PCR and nPCR

The most effective primers (S1 Table) to detect HBV sequences by first round-PCR in the control group were those that amplified the products SPI: 7/10 (70%), followed by P and PC/C: 5/10 (50%), SP: 2/10 (20%), PX, PSI and PSII: 1/10 (10%). While the most effective primers for nPCR, amplified nSP1, nP and nPC/C: 10/10 (100%), followed by nSP3: 9/10 (90%), nPX: 7/10 (70%), nPSI: 5/10 (50%), nSP2: 4/10 (40%) and nPSII: 1/10 (10%). These results are summarized in Table 1.

**Table 1:** Products obtained with first round PCR and nested PCR for the control group.

Control Group	HBsAg	Anti-HBc S/CO	HBV DNA*	SP	SPI	nSP1	nSP2	nSP3	P	nP	PX	nPX	PC/C	nPC/C	PSI	nPSI	PSII	nPSII
BD1	+	13.2	+	-	D	nD	-	nD	D	nD	-	nD	D	nD	-	nD	-	-
BD2	+	14.1	+	-	-	nD	-	nD	-	nD	-	-	-	nD	-	nD	-	-
BD3	+	14.0	+	-	D	nD	-	nD	-	nD	-	-	-	nD	-	-	-	-
BD4_cs_	+	15.5	+	D	D	nD	nD	nD	D	nD	D	nD	D	nD	D	nD	D	nD
BD5	+	11.8	+	-	D	nD	nD		-	nD	-	nD	-	nD	-	-	-	-
BD6	+	13.2	+	D	D	nD	nD	nD	D	nD	-	nD	D	nD	-	nD	-	-
BD7	+	14.2	+	-	D	nD	-	nD	-	nD	-	nD	-	nD	-	-	-	-
BD8	+	13.8	+	-	D	nD	nD	nD	-	nD	-	**-**	-	nD	-	-	-	-
BDOBI^1^_cs_	-	7.8	-	-	-	nD	-	nD	D	nD	-	nD	D	nD	-	nD	-	-
BDOBI^2^	-	-	+	-	-	nD	-	nD	D	nD	-	nD	D	nD	-	-	-	-
Percent of detection				20	70	100	40	90	50	100	10	70	50	100	10	50	10	10

* = Procleix Ultrio Plus discriminatory and confirmatory assay;

BD1-BD8 = Blood donors HBsAg (+)/Anti-HBc (+) / DNA HBV detected; BDOBI^1^ = Blood donor HBsAg (-)/Anti-HBc (+) finally identified as OBI; D = Detection of PCR products in first round and nD = Detection of nested PCR products,— = Non-reactive HBsAg or Anti-HBc and non-detection of PCR products. _cs_ = the PCR products of BD4 and BDOBI^1^ were cloned and sequenced.

### Patients

Forty-five (38.8%) of the 116 HBsAg (-)/anti-HBc (+) evaluated patients met the inclusion criteria; 45.5% were female and 54.5% male, age range was 26–84 years old, average age was 57.5 years, 13.3% had been vaccinated against HBV, 6.6% were HBeAg (+) and 40% had a diagnosis of liver disease (S2 Table).

### Efficacy of primers for the detection of the HBV genome in HBsAg (-) /anti-HBc (+) patients by nPCR

Serum samples of all the 45 HBsAg (-)/anti-HBc (+)patients were assayed in nPCR, using all the primer pairs shown in S1 Table. Twenty-nine (64.4%) of the 45 samples were positive for detection of at least two regions of the HBV genome (Table 2).

**Table 2:** Detection of HBV DNA products by nested and real-time PCR in a group of HBsAg (-)/anti-HBc (+) patients from Puebla, México.

Patients	nSP1	^rt^SP1	nSP3	^rt^SP3	nP	^rt^P	nPX	^rt^X	nPC/C	^rt^C	nPSI	^rt^PSI	nPSII	GT	OBI
1	nD^S^	^rt^D	nD	^rt^D	nD	^rt^D	-	^rt^D	nD^S^	^rt^D	-	^rt^D	-	H	Yes
2	nD^S^	^rt^D	nD	^rt^D	nD	^rt^D	-	^rt^D	nD^S^	^rt^D	-	^rt^D	-	H	Yes
3	nD^S^	^rt^D	nD^S^	^rt^D	nD^S^	-	nD^S^	^rt^D	nD^S^	^rt^D	nD	^rt^D	-	H	Yes
4	nD	^rt^D	-	^rt^D	-	^rt^D	-	^rt^D	nD^S^	^rt^D	-	-	-	H	Yes
5	nD	^rt^D	nD	^rt^D	nD^S^	^rt^D	nD	^rt^D	nD^S^	^rt^D	-	-	-	H	Yes
6	nD^S^	^rt^D	nD	^rt^D	nD	^rt^D	-	^rt^D	nD^S^	^rt^D	-	-	-	H	Yes
7	nD^S^	^rt^D	nD	^rt^D	nD^S^	^rt^D	-	^rt^D	nD^S^	^rt^D	-	-	-	H	Yes
8	nD^S^	^rt^D	nD	^rt^D	nD	^rt^D	-	^rt^D	nD^S^	^rt^D	-	-	-	H	Yes
9	nD	^rt^D	nD	^rt^D	nD	^rt^D	nD^S^	^rt^D	nD^S^	^rt^D	-	-	-	H	Yes
10	nD^S^	^rt^D	nD	^rt^D	nD	^rt^D	-	^rt^D	nD^S^	^rt^D	-	^rt^D	-	H	Yes
11	nD^S^	^rt^D	-	^rt^D	nD^S^	-	-	^rt^D	nD^S^	^rt^D	-	-	-	H	Yes
12	nD	^rt^D	nD ^S^	^rt^D	-	^rt^D	-	-	nD^S^	^rt^D	-	-	-	H	Yes
13	nD^S^	^rt^D	nD ^S^	^rt^D	-	^rt^D	-	-	nD^S^	^rt^D	-	-	-	H	Yes
14	nD^S^	^rt^D	nD	^rt^D	nD	^rt^D		^rt^D	nD^S^	^rt^D	-	-	-	H	Yes
15	nD^S^	^rt^D	nD	^rt^D	nD^S^	^rt^D	-	^rt^D	nD^S^	^rt^D	-	-	-	H	Yes
16	nD^S^	^rt^D	nD	^rt^D	nD	^rt^D	nD^S^	-	nD ^S^	^rt^D	-	^rt^D	-	H	Yes
17	nD^S^	^rt^D	nD	^rt^D	nD	^rt^D	-	^rt^D	nD ^S^	^rt^D	-	^rt^D	-	H	Yes
18	nD^S^	^rt^D	nD^S^	^rt^D	nD	^rt^D	-	-	-	^rt^D	-	-	-	H	Yes
19	nD	^rt^D	nD	^rt^D		^rt^D	-	^rt^D	nD ^S^	^rt^D	-	^rt^D	-	H	Yes
20	nD^S^	^rt^D	nD^S^	^rt^D	nD	^rt^D	-	^rt^D	-	^rt^D	nD^S^	^rt^D	-	H	Yes
21	nD^S^	^rt^D	nD	^rt^D	-	^rt^D	-	^rt^D	nD ^S^	^rt^D	-	-	-	H	Yes
22	nD^S^	^rt^D	nD	^rt^D	-	-	-	-	-	^rt^D	nD^S^	^rt^D	-	H	Yes
23	nD^S^	^rt^D	nD	-	-	^rt^D	nD	^rt^D	nD^S^	^rt^D	-	-	-	H	Yes
24	nD^S^	^rt^D	nD	^rt^D	nD^S^	^rt^D	-	^rt^D	-	^rt^D	-	-	-	H	Yes
25	nD^S^	^rt^D	nD	^rt^D	nD^S^	^rt^D	-	^rt^D	-	^rt^D	-	-	-	H	Yes
26	nD^S^	^rt^D	nD	^rt^D	-	^rt^D	-	-	-	^rt^D	-	-	-	H	Yes
27	nD^S^	^rt^D	nD	^rt^D	-	^rt^D	-	^rt^D	-	^rt^D	-	-	-	H	Yes
28	nD^S^	^rt^D	nD^S^	^rt^D	nD^S^	^rt^D	nD^S^	^rt^D	nD	^rt^D	-	-	-	H	Yes
29	-	^rt^D	-	^rt^D	-	^rt^D	-	^rt^D	-	^rt^D	nD	^rt^D	nD^S^	H	Yes
30	-	^rt^D	-	^rt^D	-	-	-	^rt^D	-	^rt^D^S^	-	-	-	H*	Yes
31	-	^rt^D	-	^rt^D	-	-	-	^rt^D	-	^rt^D^S^	-	-	-	H*	Yes
32	-	^rt^D	-	-	-	^rt^D	-	^rt^D^S^	-	^rt^D	-	-	-	H*	Yes
33	-	^rt^D	-	^rt^D	-	^rt^D	-	^rt^D^S^	-	^rt^D	-	-	-	H*	Yes
34	-	^rt^D	-	^rt^D	-	^rt^D	-	-	-	^rt^D	-	^rt^D^S^	-	H*	Yes
35	-	^rt^D	-	-	-	-	-	^rt^D	-	^rt^D	-	-	-	NG	No
36–38	-	^rt^D	-	-	-	-	-	^rt^D	-	-	-	-	-	NG	No
39–45	-	-	-	-	-	-	-	-	-	-	-	-	-	NG	No
%	62.2	84.4	57.8	71.1	42.2	64.4	13.3	71.1	46.7	77.8	11.1	24.4	2.2	100	75.5

n = nested PCR, ^rt^ = real time PCR; nD^S^ or ^rt^D^S^ = HBV DNA detected and sequenced (concentration >15ng/μl), nD or ^rt^D = HBVDNA detected but not sequenced due to low concentration,— = HBV DNA not detected in this region,

* = genotyped with the real-time PCR product, NG = Not genotyped, OBI = Occult hepatitis B virus infection.

The most effective primer pairs to detect HBV sequences by nPCR were: nSP1s-nSP1a: 28/45 (62.2%), nSP3s-nSP3a: 26/45 (57.8%), nPC/Cs-nPC/Ca: 21/45 (46.7%) and nPs-nPa: 19/45 (42.2%); while the least effective primer pairs were nPXs-nPXa: 6/45(13.3%), nPSIs-nPS1a: 4/45 (8.8%) and nPSIIs-PSIa: 1/45 (2.2%). The primer pair nSP2s-nSP2a did not generate amplicons in any case (Table 3). The effectiveness of primer pairs to obtain nPCR products was higher in the control group than in the HBsAg (-)/anti HBc (+) patient group (Table 3).

**Table 3:** Effectiveness of primer pairs to detect HBV DNA in HBsAg (-)/anti-HBc (+) patients from Puebla Mexico by nested PCR.

Primer pairs	HBV genome region ORF	Control group n (%)	HBsAg (-)/Anti-HBc (+) patients n (%)
nSP1s nSP1a	PreS_2_/P S/P	10 (100)	28 (62.2)
nSP3s nSP3a	PreS_2_/P S/P	9 (90)	26 (57.7)
nPC/Cs nPC/Ca	X/PreC C	10 (100)	21 (46.6)
nPs nPa	P P/X	10 (100)	19 (42.2)
nPXs nPXa	P/X X	7 (70)	6 (13.3)
nPSIs nPSIa	C/P P	5 (50)	3 (8.8)
nPSIIs PSIa	P PreS_1_/P	1(10)	1 (2.2)
nSP2s nSP2a	S/P P	4 (40)	0 (0.0)

### Efficacy of primers for the detection of the HBV genome in HBsAg (-)/anti-HBc (+) patients by real time PCR

Of the 45 HBsAg (-)/anti-HBc (+) serum samples, 38 (84.4%) were positive for amplification of some region of the HBV genome with primers for real time PCR (S1 Table). The 6 products obtained (^rt^SP1, ^rt^SP3, ^rt^P, ^rt^C, ^rt^X and ^rt^PSI) are shown in agarose gel (S1 Fig).

The most effective primer pairs to detect HBV products by real- time PCR were: SP1s-SP1a: 38/45 (84.4%), Cs-Ca: 35/45 (77.8%), SP3s-SP3a 32/45 (71.1%), Xs-Xa: 31/45 (68.8%) and Pols-Pola: 29/45 (64.4%); while the least effective pair was PolSIs-PolSIa: 11/45 (24.4%) (Table 4).

**Table 4:** Effectiveness of primer pairs to detect HBV DNA in HBsAg (-)/anti-HBc (+) patients from Puebla, Mexico by real-time PCR.

Primer pair	HBV genome region ORF	HBsAg (-)/Anti HBc (+) Patients ^rt^PCR (+) (%)	Rate of ^rt^PCR (+) in nPCR (-) samples n^rtPCR (+)^/n^nPCR(-)^ (%)	Rate of in ^rt^PCR (-) in nPCR (+) samples n^rtPCR (-)^ / n^nPCR(+)^ (%)
SP1s SP1a	PreS_2_/P S/P	38 (84.4)	10 /17 (58.8)	0/28 (0.0)
Cs Ca	C C	35 (77.8)	14/24 (58.3)	0/21 (0.0)
SP3s SP3a	S/P S/P	32 (71.1)	7/19 (36.8)	1/26 (3.8)
Xs Xa	X X	31 (68.8)	26/39 (69.2)	1/6 (16.6)
Pols Pola	P P	29 (64.4)	12/26 (38.4)	2/19 (10.5)
PolSIs PolSIa	P P	11 (24.4)	7/41 (17.0)	0/4 (0.0)

### Patients who met the definition of occult hepatitis B virus infection

Of the initial 45 probable occult hepatitis B patients that were HBsAg (-)/anti-HBc (+), 34 (75.5%) had HBV detected by PCR in at least three different viral genomic regions and thus met the criteria to be considered with occult hepatitis B virus infection. Of them, 29 (64.4%) were detected both by nPCR and real-time PCR, while 5 more (11.1%) were detected by real-time PCR only (Table 2). The sequenced products of OBI patients and control group were analyzed and 100% of individuals in both groups had genotype H (Tables 1 and 2). Four patients were DNA HBV positive by real time PCR but were not considered as OBI because one of them was positive for three different segments by real-time PCR, but none was successfully sequenced; the other three patients were positive in only two distinct real-time PCR assays and none could not be sequenced (Table 2). Seven samples were negative in nPCR and real time PCR and were then considered not to be infected by HBV (Table 2).The effectiveness of primer pairs to detect the HBV DNA in different types of samples were arranged from highest to lowest and summarized in Tables 3 and 4.

## Discussion

HBV virions present in the serum of infected individuals have an unusually structured genome, composed of a partially double-stranded, relaxed circular DNA (rcDNA), with none of the strands covalently closed [15] and a gap in the minus strand [16]. In contrast, inside infected cells the stable form of the viral genome is a covalently closed circular DNA (cccDNA) that forms a mini-chromosome in the cell nucleus [17]. This cccDNA is found in serum in much smaller quantities than rcDNA adding complexity to the detection of the HBV genome in human serum, as not all genome regions are equally accessible in rcDNA.

To detect OBI efficiently, primers need to be designed for highly conserved sections of the HBV genotypes that circulate in each geographical region and used in highly sensitive and specific methods like nPCR or real-time PCR [3, 18]. The primers for this study were designed using the complete HBV genome sequences reported in GenBank, mainly from Mexico. The most effective primer pairs to identify the HBV genome in our HBsAg (-)/anti-HBc (+) patients, hybridized at the PreS_2_/P, S/P regions and were pairs nSP1s-nSP1a and nSP3s-nSP3a (Table 3). Recently, primers close to this region have been used to detect OBI in genotypes different than H [19–22]. We also obtained satisfactory results with primers located at the X/PreC and C regions (nPC/Cs-nPC/Ca) (Table 3), flanking the X/PreC region (nt1753 to nt1777), which is associated with substitutions and deletions found in OBI patients in other genotypes [23]. These primers also flank the gap region close to direct repeat 1 (DR1). Primers close to this region have been used previously to detect OBI [19, 24–25].

Primers that hybridize at the P/X region (nPXs-nPXa) were much less effective to produce PCR amplicons (Table 3), probably because their target sequence is within direct repeat 2 (DR2), between nt 1590 and nt 1600, very close to the single-stranded region at the 5’end of the rcDNA [15–16]. Accordingly, the nPs-nPa primers that target a similar area in the P/X region but do not include the DR2, had a better efficacy, amplifying 100% of controls and 42.2% of patients (Table 3).

PCR products with the nPSIs-nPSIa pair were found at a relatively low frequency in patients. By nested PCR, these primers produced an amplicon in 50% of controls but only 8.8% patients (Table 3). These primers hybridize at the P gene, which may be difficult to copy in its rcDNA form due to limited access. In a similar case, the nSP2s-nSP2a primers were unable to detect the HBV genome in HBsAg (-)/anti-HBc (+) patients but detected 40% of control individuals (Table 3). Sequence variation in these regions or low HBV DNA in patient samples could be involved in the different detection rate between controls and patients.

The nPSIIs-PSIa primers had low efficiency in both controls and patients, with only one positive sample in each group (Table 3). The PSIa primer is located at the PreS_1_ region, where a deletion at nt2983-3201 has been reported in OBI patients [23, 26–27] and deletions have been reported in patients in different stages of the HBV infection [28].

As expected, more patients were detected by real-time PCR than by nPCR (Table 2). More than half of nPCR negative samples were detected as positive using real-time PCR primers targeted to the PreS_2_/P, S/P and C regions (Table 4). However, the most effective real-time PCR primers to detect HBV genome in negative nPCR samples were those designed for the X region, where there is no overlap with other ORFs (Table 4). In summary, the most effective primers to detect OBI in HBsAg (-)/anti-HBc (+) patients, hybridized at the PreS_2_/P, S/P, C and X regions, which harbor the most conserved sequences.

Infrequent negative results for real-time PCR in patients that had positive nPCRs were found for primer pairs Xs-Xa in 16.6% of samples, Pols-Pola in 10.5% and SP3s-SP3a in 3.8% (Table 2). These false negative results in real time PCR were detected thanks to the simultaneous use of two PCR techniques and have not been reported before.

Under identical conditions, the Power SYBR Green PCR Master Mix was able to detect the HBV genome in more samples than the Maxima SYBR Green/ROX Master Mix; this observed in particular for primer pairs Xs-Xa and SP3s-SP3a in 50% and 20% respectively, of the ^rt^PCR (+) samples that were nPCR (-) (Table 4).

Some patients in the present study had a history of anti-HBV vaccination; however vaccination was less frequent in individuals ultimately classified as OBI (5.8% *vs* 36%). Accordingly, a recent study showed that OBI was more frequent in unvaccinated Mexican children [29]. In turn, HBeAg was detected in only 8.8% of the patients, consistent with a recent study, which proposed that OBI with positive HBeAg is rare and associated with active HBV replication [30]. HBeAg has been previously detected in Mexican blood donors with OBI at a low frequency (4%) [31].

A high percentage of HBsAg (-)/anti-HBc (+) of patients with (87.5%) and without (66.6%) liver disease were detected as OBI in our study, similar to previously reported in [3] and [19], respectively. In Mexico, OBI has been detected in 14.2% of native population (Nahuas and Huichol) only reactive to anti-HBc and without a diagnosis of liver disease [32]. In mexican blood donors it has been detected ranging from 11 to 17.3% [31,33], and in HIV-1 patients in 49% [24]. However, more studies on this health problem are needed to determine the true impact of OBI in the Mexican population. The strategy and sets of primers described here can improve HBV and OBI detection protocols.

## Conclusions

In this study, we have proposed and tested sets of primers for the detection of the HBV genome by nPCR and real-time PCR in probable OBI patients. Primers designed for the PreS2/P, S/P, C and X regions were able to confirm 75.5% of probable OBI cases.

## Supporting information

S1 Fig Example of the six real time PCR products obtained with the primers listed in S1 Table. (DOCX)

S1 Table Primers to detect hepatitis B virus in serum of anti-HBc (+)/HBsAg (-) patients by PCR (first round), nested PCR, and real time PCR. (DOCX)

S2 Table Data of anti-HBc (+)/HBsAg (-) patients from Puebla, México. (DOCX)

S3 Table Sequenced ^rt^PCR products that were too short to upload to GenBank and their viral genotype as identified through a GenBank blast. (DOCX)
