# Supplementary material for: Effectiveness of PCR primers for the detection of occult hepatitis B virus infection in Mexican patients
Source: PLoS One. 2018 Oct 10;13(10):e0205356. doi: 10.1371/journal.pone.0205356 (PMC6179258; doi:10.1371/journal.pone.0205356)
Supplement: S1 Fig — (DOCX) [file pone.0205356.s001.docx]

4

8

7

6

5

3

2

1


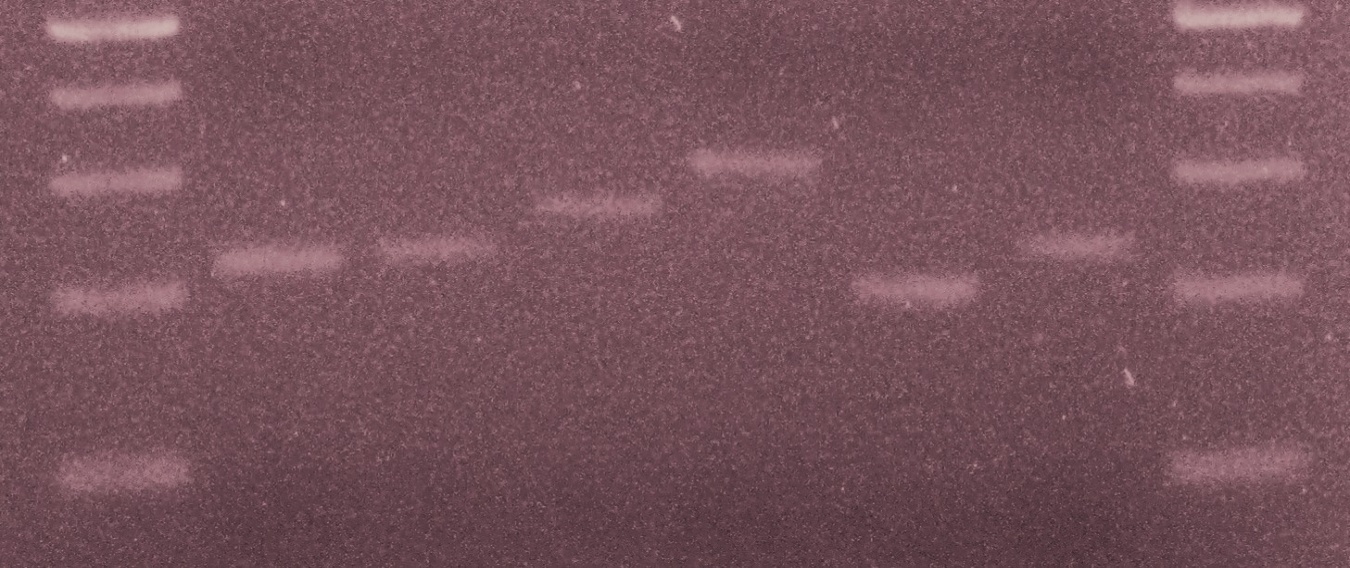


100 bp

150 bp

50 bp

150 bp

50 bp

100 bp

117 bp

100 bp

153 bp

130 bp

115 bp

110 bp

**S1 Fig.** Example of the six real time PCR products obtained with the primers listed in S1 Table (Amplified from control samples): Lane 1 50 bp marker, lane 2  ^rt^SP1 (110 bp), lane 3  ^rt^SP3 (115 bp), lane 4 ^rt^P (130 bp), lane 5 ^rt^X (153 bp), lane 6 ^rt^C (100 bp), lane 7 ^rt^PSI (117 bp), lane 8 50bp marker..
