# Supplementary material for: Effectiveness of PCR primers for the detection of occult hepatitis B virus infection in Mexican patients
Source: PLoS One. 2018 Oct 10;13(10):e0205356. doi: 10.1371/journal.pone.0205356 (PMC6179258; doi:10.1371/journal.pone.0205356)
Supplement: S1 Table — (DOCX) [file pone.0205356.s002.docx]

**S1 Table.** Primers to detect hepatitis B virus in serum of anti-HBc (+)/HBsAg (-) patients by PCR (first round), nested PCR, and real time PCR.

| Name and sequence | | Position* | Annealing  (°C) | | Product  name (size) |
| --- | --- | --- | --- | --- | --- |
| PCR first round | | | | | |
| PreS_2_s  SP.a. | 5´-TGCTTCCACCAATCGGAGGTCA-3´  5´-ACAGGCATACAAGGGCATGAGAG-3´ | 3129-3150  1071-1048 | 56-60 | | SP(1158) |
| PreS_2_s  NF2a | 5´-TGCTTCCACCAATCGGAGGTCA-3´  5´-ACTGAGCCAAGAGAAACGGGCT-3´ | 3129-3150  676-655 | 56-60 | | SPI(763) |
| Ps  Pa | 5´-GGTTATCCTGCTYTMATGCC-3´  5´-GTTCACGGTGGTCTCCATGC-3´ | 1039-1058  1625-1606 | 56-60 | | P(587) |
| PXs  PXa | 5´-GTGCTGCCAACTGGATCCTG-3´  5´-CAAGGCACAGCTTGGAGGCTTG-3´ | 1388-1407  1886-1859 | 57 | | PX(499) |
| PC/Cs  PC/Ca | 5´-GGAGGCTGTAGGCATAAATTGG-3´  5´-CCCACCTTATGAGTCCAAGG-3´ | 1776-1796  2476-2457 | 50-54 | | PC/C(702) |
| PSIs  PSIa | 5´-CCCTCGCCTCGCAGACGAAG-3´  5´-TCCACWGCATTGCCTGKGGATG-3´ | 2381-2400  3214-3193 | 46 | | PSI(834) |
| PSIs  SP_ccc_a | 5´-CCCTCGCCTCGCAGACGAAG-3´  5´-TGAACTGGAGCCACCAGCAG-3´ | 2381-2400  76-57 | 52 | | PSII(910) |
| NestedPCR | | | | | |
| nSP1s  nSP1a | 5´-CTCAACACAGTTCCACCAAGCACTG-3´  5´-AGACACATCCAACGATAGCCAGGAC-3´ | 1-25  384-360 | 56-60 | nSP1(384) | |
| nSP2s  nSP2a | 5´-TGTCCTGGCTATCGTTGGATGTGT-3´  5´-CCACAATGCGTTGACAAACCTTCCA-3´ | 359-384  1003-979 | 56-60 | nSP2(645) | |
| nSP3s  nSP3a | 5´-CTGCTCCGACTATTGCCTCTCTCAC-3´  5´-GTGCTGGTGGTTGTAGATCCTGGA-3´ | 90-114  507-484 | 56-60 | nSP3(418) | |
| nPs  nPa | 5´-TGCCAAGTGTTTGCTGACGCAAC-3´  5´-CGACGGGACGTAGACAAAGGAC-3´ | 1174-1196  1437-1416 | 62 | nP(264) | |
| nPXs  nPXa | 5´-ACTTCGCTTCACCTCTGCACG-3´  5´-CATGGTGCTGGTGAACAGACCA-3´ | 1583-1603  1816-1795 | 53 | nPX(234) | |
| nPC/Cs  nPC/Ca | 5´-TCTGTTCACCAGCACCATGCA-3´  5´-TGGTGGTCTGTAAGCAGGTGGA-3´ | 1798-1818  2305-2284 | 58 | nPC/C (508) | |
| nPSIs  nPSIa | 5´-TCTCAATCACCGCGTCGCA-3´  5´-GCTGTAGCTCTTGTTCCCAGGA-3´ | 2402-2420  2847-2826 | 52 | nPSI(446) | |
| nPSIIs  PSIa | 5´-TCCTGGGAACAAGAGCTACAGC-3´  5´-TCCACWGCATTGCCTGKGGATG-3´ | 2826-2847  3214-3193 | 52 | nPSII(389) | |
| Real-timePCR | | | | | |
| SP1s  SP1a | 5´-GCTCCGACTATTGCCTCTCTCACA-3´  5´-TGTAACACGAGAAGGGGTCCTAGGA-3´ | 92-115  201-177 | 60-65 | | ^rt^SP1(110) |
| SP3s  SP3a | 5´-ACTCGTGGTGGACTTCTCTCAA-3´  5´-CGAGACAAGTTGGAGGACAAGAGA-3´ | 252-273  366-342 | 60 | | ^rt^SP3(115) |
| Pols  Pola | 5´-TGTCTCCTCTGCCGATCCATACTG-3´  5´-AGCCATGGGAAGGAGGTGTACT-3´ | 1250-1273  1379-1358 | 60-65 | | ^rt^P(130) |
| Xs  Xa | 5´-CCTTACATAAGAGGACTCTTGGAC-3´  5´-AGACCAYTTTATGCCTACAGC-3´ | 1648-1671  1800-1780 | 60 | | ^rt^X(153) |
| Cs  Ca | 5´-CAGAGATGCCTTAGAATCACCCGAAC-3´  5´-CCAGGAAGCCAAAGTCATCAACTCAC-3´ | 2014-2039  2113-2088 | 60-65 | | ^rt^C(100) |
| PolSIs  PolSIa | 5´-GGTCTTTACTCCTCTACTGTACCTM-3´  5´-GAGTGGGCCTACAMMTTGYTYACA-3´ | 2487-2511  2603-2580 | 60 | | ^rt^PSI(117) |

The position number of the nucleotides corresponds to the complete HBV genome sequence deposited in the GenBank (NCBI) with accession number AB516395 (genotype H).
