# Supplementary material for: Effectiveness of PCR primers for the detection of occult hepatitis B virus infection in Mexican patients
Source: PLoS One. 2018 Oct 10;13(10):e0205356. doi: 10.1371/journal.pone.0205356 (PMC6179258; doi:10.1371/journal.pone.0205356)
Supplement: S2 Table — (DOCX) [file pone.0205356.s003.docx]

**S2 Table.** Data of anti-HBc (+)/HBsAg (-) patients from Puebla, México.

| Data of patients | | | | Serologic markers | | | Diagnostic |
| --- | --- | --- | --- | --- | --- | --- | --- |
| *Patients | Gender | Age  (years) | Vaccinated | HBsAg | Anti-HBc  S/CO | HBeAg | Liver disease |
| 1 | F | 56 | NO | NR | 10.7 | NR | YES |
| 2 | M | 63 | NO | NR | 7.51 | NR | YES |
| 3 | M | 62 | NO | NR | 7.75 | NR | NO |
| 4 | F | 61 | NO | NR | 12.2 | NR | YES |
| 5 | F | 49 | NO | NR | 12.1 | R | YES |
| 6 | F | 61 | NO | NR | 4.1 | NR | YES |
| 7 | M | 44 | NO | NR | 3.6 | NR | YES |
| 8 | M | 84 | NO | NR | 7.7 | NR | NO |
| 9 | F | 61 | NO | NR | 8.6 | NR | NO |
| 10 | F | 56 | NO | NR | 4.4 | NR | NO |
| 11 | F | 61 | NO | NR | 6.8 | NR | YES |
| 12 | M | 66 | NO | NR | 4.0 | NR | YES |
| 13 | M | 59 | NO | NR | 2.6 | R | YES |
| 14 | F | 62 | NO | NR | 10.6 | NR | NO |
| 15 | M | 65 | NO | NR | 9.9 | NR | NO |
| 16 | M | 57 | NO | NR | 10.4 | NR | YES |
| 17 | F | 61 | NO | NR | 8.2 | NR | YES |
| 18 | F | 63 | NO | NR | 6.8 | NR | NO |
| 19 | M | 55 | NO | NR | 12.1 | NR | NO |
| 20 | M | 65 | NO | NR | 8.3 | NR | NO |
| 21 | M | 57 | NO | NR | 8.2 | NR | YES |
| 22 | F | 59 | NO | NR | 3.2 | NR | YES |
| 23 | F | 61 | NO | NR | 2.6 | NR | NO |
| 24 | F | 55 | NO | NR | 11.6 | NR | NO |
| 25 | M | 63 | NO | NR | 7.2 | NR | NO |
| 26 | M | 57 | NO | NR | 7.75 | NR | NO |
| 27 | F | 63 | NO | NR | 2.2 | NR | NO |
| 28 | M | 58 | NO | NR | 8.3 | R | YES |
| 29 | M | 80 | NO | NR | 7.0 | NR | NO |
| 30 | M | 56 | NO | NR | 8.0 | NR | NO |
| 31 | M | 53 | NO | NR | 5.7 | NR | YES |
| 32 | F | 53 | NO | NR | 13.6 | NR | NO |
| 33 | M | 31 | YES | NR | 10.6 | NR | YES |
| 34 | M | 33 | YES | NR | 11.0 | NR | NO |
| 35 | F | 45 | NO | NR | 6.7 | NR | NO |
| 36 | F | 57 | NO | NR | 4.3 | NR | NO |
| 37 | M | 41 | YES | NR | 2.0 | NR | YES |
| 38 | F | 45 | NO | NR | 9.6 | NR | NO |
| 39 | M | 68 | NO | NR | 5.9 | NR | NO |
| 40 | M | 54 | NO | NR | 10.6 | NR | NO |
| 41 | F | 56 | YES | NR | 4.7 | NR | NO |
| 42 | M | 76 | NO | NR | 11.1 | NR | NO |
| 43 | M | 26 | YES | NR | 11.4 | NR | NO |
| 44 | M | 38 | YES | NR | 2.44 | NR | YES |
| 45 | M | 69 | NO | NR | 4.9 | NR | NO |
|  | F 20 (45.5%)  M 25 (54.5%) | Range  26 - 84  Average (95%CI)  57.5(53.7 – 61.1) | 6(13.3%) | Reactive  0 (0.0%) | Range  2.0 -13.6  Average (95%CI)  7.52 (6.54 -8.51) | Reactive  3(6.6%) | With liver disease  18(40%) |

*Patients with inclusion criteria; R= reactive, NR = non-reactive; S/CO = Sample RLU (relative light unit) /calibrator RLU; HBsAg

reactive =S/CO >1.0; Anti-HBcreactive = S/CO >1.0; HBeAg reactive = ≥COV. COV = The averageOD_450_ of negative control x factor.
