# Supplementary material for: Effectiveness of PCR primers for the detection of occult hepatitis B virus infection in Mexican patients
Source: PLoS One. 2018 Oct 10;13(10):e0205356. doi: 10.1371/journal.pone.0205356 (PMC6179258; doi:10.1371/journal.pone.0205356)
Supplement: S3 Table — (DOCX) [file pone.0205356.s004.docx]

**S3 Table.** Sequenced ^rt^PCR products that were too short to upload to GeneBank and their viral genotype as identified through a Gene Bank Blast.

| **Patient 30** |
| --- |
| Primer: Cs 5´-_2014_CAGAGATGCCTTAGAATCACCCGAAC_2039_-3´  Ca 5´-_2113_CCAGGAAGCCAAAGTCATCAACTCAC_2088_-3´ |
| Product: ^rt^C without primers  _2040_ATTGCTCCCCCCACCATACTGCTCTCAGGCAATCTATTTGCTGCTGGG_2087_ |
| Blast: Identity 96% core protein, accession KY775673.1, genotype H |
|  |
| **Patient 31** |
| Primer: Cs 5´-_2014_CAGAGATGCCTTAGAATCACCCGAAC_2039_-3´  Ca 5´-_2113_CCAGGAAGCCAAAGTCATCAACTCAC_2088_-3 |
| Product: ^rt^C without primers  _2040_ATTGCTCCCCCCACCATACTGCTCTCAGGCAATCTATTTGCTGCTGGG_2087_ |
| Blast: Identity 96% core protein, accession KY775673.1, genotype H |
|  |
| **Patient 32** |
| Primers Xs 5´-_1648_CCTTACATAAGAGGACTCTTGGAC_1671_-3´  Xa 5´-_1800_AGACCAYTTTATGCCTACAGC_1780_-3´ |
| Product: ^rt^X without primers  _1672_TTTCGCCCCGGTCAACGACCTGGATTGAGGACTACATCAAAGACTGTGTATTTAAGGACTGGGAGGAGTCAGGGGAGGAGTTGAGGTTAAAGGTCTTTGTATTAGGAG_1779_ |
| Blast: Identity 100% X protein, accession HM066946.2, genotype H |
|  |
| **Patient 33** |
| Primers Xs 5´-_1648_CCTTACATAAGAGGACTCTTGGAC_1671_-3´  Xa 5´-_1800_AGACCAYTTTATGCCTACAGC_1780_-3´ |
| Product: ^rt^X without primers  **_16_**_72_TTTCGCCCCGGTCAACGACCTGGATTGAGGACTACATCAAAGGCTGTGTATTTAAGGACTGGGAGGAGTCAGGGGAGGAGTTGAGGTTAAAGGTCTTTGTATTAGGAG_1779_ |
| Blast: Identity 99% X protein, accession HM066946.2, genotype H |
|  |
| **Patient 34** |
| Primers PolSIs 5´-_2487_GGTCTTTACTCCTCTACTGTACCTM_2511_-3´  PolSIa 5´-_2603_GAGTGGGCCTACAMMTTGYTYACA_2580_-3´ |
| Product: ^rt^PSI without primers  _2521_CTGACTGGCTAACTCCTTCTTTTCCTGACATTCACTTGCATCAAGATCTGATACAAAAA_2579_ |
| Blast: Identity 100% polymerase, accession KY595544.1, genotype H |
